# Supplementary material for: Senescent cells secrete chromatin components via senescence-associated extracellular particles
Source: Res Sq. 2026 Jan 12:rs.3.rs-8349343. Preprint. [Version 1] doi: 10.21203/rs.3.rs-8349343/v1 (PMC12869639; doi:10.21203/rs.3.rs-8349343/v1)

Supplemental Figure Legends:

Supplemental Figure 1: **Validation and characterization of CCF-specific secretion.**

(**A**) Representative immunofluorescence images of proliferating cells treated with DMSO and senescent cells treated with the inhibitor of CCF formation, MDM2 inhibitor RG7388 (MDM2i) or DMSO vehicle, demonstrating decreased CCFs in the treated cells[20].

(**B**) Quantification of the number of CCFs per cell in proliferating cells treated with DMSO and senescent cells treated with MDM2i or DMSO vehicle. The y-axis represents the total number of CCF, normalized to the total number of nuclei. Data are presented as mean ± SD (n=3 independent experiments). Statistical significance was determined using two-sided one-way ANOVA: ***p=0.0003.

(**C**) Schematic representation of centrifugation steps to isolate large extracellular vesicles (EVs), small extracellular vesicles and particles (sEVPs), and soluble proteins. Conditioned media collected from IMR90 cells cultured in FBS-free medium were subjected to differential centrifugation to sequentially remove cells and apoptotic bodies, then large shedding vesicles. The resulting cleared medium was ultracentrifuged at 100,000 × g to pellet crude sEVPs, which were resuspended in PBS and ultracentrifuged again at 100,000 x g to eliminate residual conditioned media and obtain an enriched sEVP fraction. The remaining sEVP-depleted conditioned media were processed by TCA protein precipitation to analyze the soluble protein fraction.

Supplemental Figure 2: Senescent cells upregulate the set of genes involved in endosomal pathways.

(**A**) Analysis of four previously published RNA-seq data sets derived from multiple models of senescence in IMR90 cells, including replicative senescence (RS), oncogene-induced

senescence (OIS), and irradiation-induced senescence (IRS)[16,20], comparing proliferating control versus senescent. Heatmaps display differentially expressed genes associated with the endosomal pathway across the four datasets. In each heatmap, red indicates higher expression and blue indicates lower expression relative to the corresponding proliferating controls.

(**B**) Venn diagrams illustrating the overlap of endosomal pathway genes that are consistently upregulated (left) or downregulated (right) across all four RNA-seq data sets corresponding to distinct models of senescence in IMR90 cells (RS, OIS, and IRS). Only genes meeting the criteria for significant differential expression relative to proliferating controls in each dataset were included.

Supplemental Figure 3: Blocking intracellular degradation of CCFs by ATG16L1 knockout upregulates secretion of the components of CCFs.

(**A**) Representative western blot of indicated proteins in whole cell lysates (WCL) and the isolated small extracellular vesicles and particles (sEVP) fractions from conditioned media of senescent wild type (WT) and senescent ATG16L1 knockout (KO) IMR90 cells treated with an inhibitor of the ESCRT-independent pathway, GW4869, and/or DMSO vehicle. GW4869 treatment was applied only during the final 24 h of the conditioning period. WCL samples were normalized to total cell number, and equal amounts of cell lysates were loaded for each condition. The isolated sEVP fractions were resuspended in identical volumes of sample buffer, and equal volumes were loaded per condition. (n=3 independent experiments).

(**B**) Quantification of the number of CCFs in proliferating and senescent WT or ATG16L1 knockout IMR90 cells treated with DMSO and/or GW4869. GW4869 treatment was applied only during the final 24 h of the conditioning period. The y-axis represents the total number of CCF, normalized to the total number of nuclei. Data are presented as mean ± SD (n=3 independent experiments).

Statistical significance was determined using two-sided one-way ANOVA: *p=0.045, *p=0.032, *p=0.019.

Supplemental Figure 4: Senescence alters the secretion profile of small extracellular vesicles and extracellular particles across cellular models, perturbation, and aging.

(**A**) Representative plots of dynamic light scattering (DLS) analysis of the small extracellular vesicles and particles (sEVP) fractions from proliferating cells treated with DMSO and senescent IMR90 cells treated with lysosomal inhibitor Bafilomycin A (BafA) or DMSO vehicle, showing size distribution (diameter, nm) and number (%), representing the estimated fraction of particle number. BafA treatment was applied only during the final 24 h of the conditioning period. The isolated small extracellular vesicles and particles (sEVP) fractions were resuspended in identical volumes of sample buffer, and equal volumes were loaded per condition.

(**B**) Quantification of size distribution determined by DLS of secreted sEVPs from proliferating cells treated with DMSO and senescent IMR90 cells treated with BafA or DMSO vehicle. BafA treatment was applied only during the final 24 h of the conditioning period. The isolated sEVP fractions were resuspended in identical volumes of sample buffer, and equal volumes were loaded per condition. Data are presented as mean ± SD (n=3 independent experiments). Statistical significance was determined using two-sided unpaired one-way ANOVA: ns.

(**C**) Quantification of the number of detected sEVPs (kilo count per second) secreted by proliferating cells treated with DMSO and senescent IMR90 cells treated with BafA or DMSO vehicle. BafA treatment was applied only during the final 24 h of the conditioning period. The isolated sEVP fractions were resuspended in identical volumes of sample buffer, and equal volumes were loaded per condition. Data are presented as mean ± SD (n=3 independent experiments). Statistical significance was determined using two-sided one-way ANOVA: *p =0.016, ****p<0.0001.

(**D**) Representative histograms of the single vesicle flow cytometry (vFC) analysis of the isolated sEVP fractions secreted by proliferating cells, senescent cells, and senescent cells treated with BafA and/or DMSO vehicle. The sample was stained with the fluorogenic membrane dye vFRed to label and size membrane particles and with a mix of anti-CD9, CD63, and CD81 antibodies conjugated to phycoerythrin (PE) to stain these common tetraspanins.

The top panel presents two-parameter histograms of estimated membrane particle diameter versus PE fluorescence, calibrated in units of antibody per cell. The gate for PE positivity was set at the 99th percentile of particle background signal (LOD ~30 ABC units), and the number and percent of particles exceeding this threshold, as well as their median brightness, are indicated. Events to the right of the vertical gate represent PE-high, tetraspanin-positive particles (canonical sEVs expressing CD9/CD63/CD81), whereas events to the left of the gate represent PE-labeled lipid particles lacking detectable tetraspanins (tetraspanin-negative particles). The bottom panels show the corresponding single-parameter histograms for samples stained with (white) or without antibody (grey).

(**E**) Summary of sEVP concentrations for tetraspanin-negative (sEVPs) and tetraspanin-positive (sEVPs+TS) populations within sEVP fractions isolated from conditioned media of proliferating cells treated with DMSO and senescent cells treated with BafA or DMSO vehicle. Buffer-only controls, synthetic liposomes (Lipo100), and platelet-derived sEV standards (PLT sEVs) were included as reference samples. Data represent n = 2 experimental replicates, with two technical replicates per condition (except controls).

(**F**) Size exclusion chromatography (SEC) column to separate components of the sEVP fraction based on their size. TEM representative images of the separated sEVP fractions (fractions 7 and 8) secreted by senescent cells using a SEC column, demonstrating pseudoparticles present in fractions 7 and 8 (n=3 independent experiments).

(**G**) Representative TEM images of the sEVP fractions isolated from IMR90 cells induced into senescence by replication RS and OIS, demonstrating the presence of extracellular particles. sEVPs were isolated from equivalent numbers of cells for each condition and resuspended in identical buffer volumes prior to downstream analyses (n=3 independent experiments).

(**H**) Representative TEM images of the sEVP fractions isolated from proliferating, RS and IRS melanocytes. sEVPs were isolated from equivalent numbers of cells for each condition and resuspended in identical buffer volumes prior to downstream analyses (n=3 independent experiments).

(**I**) Representative TEM images of isolated sEVP fractions from the plasma of young (4 months) and old mice (21 months). Equal volumes of plasma were collected from each mouse and pooled within age groups (young vs. old) prior to sEVP isolation. Isolated sEVPs were resuspended in identical buffer volumes before imaging (n = 3 independent experiments).

(**J**) Quantification of sEVs and extracellular particles secreted in the plasma of young (4 months) and old (21 months) mice. Equal volumes of plasma were collected from each mouse and pooled within age groups (young vs. old) prior to sEVP isolation. Isolated sEVPs were resuspended in identical buffer volumes before imaging. Data are presented as mean ± SD (n = 3 independent experiments). Statistical significance was determined using a two-sided t-test: **p =0.0029.

Supplemental Figure 5: **Autophagy inhibition alters the secretion profile of senescent cells, enhancing the release of extracellular particles and associated dsDNA.**

(**A**) TEM representative images of the isolated small extracellular vesicles and particles (sEVP) fractions secreted by wild-type (WT) proliferating and senescent cells or ATG16L1 knockout (KO) proliferating and senescent cells treated with with an inhibitor of the ESCRT-independent pathway, GW4869 or DMSO. GW4869 treatment was applied only during the final 24 h of the

conditioning period. sEVPs were isolated from equivalent numbers of cells for each condition and resuspended in identical buffer volumes prior to imaging.

(**B**) Quantification of the isolated sEVs and extracellular particles secreted by WT or ATG16L1 knockout proliferating or senescent cells treated with GW4869 or DMSO vehicle. GW4869 treatment was applied only during the final 24 h of the conditioning period. sEVPs were isolated from equivalent numbers of cells for each condition and resuspended in identical buffer volumes prior to imaging. Data are presented as mean ± SD (n = 3 independent experiments). Statistical significance was determined using two-sided one-way ANOVA: for sEVs/field: * p =0.0132 (control proliferating vs control senescent), 0.0118 (control senescent +/- GW4869 treatment), **p=0.0012 (knockout senescent +/- GW4869 treatment); for extracellular particles/field: **p=0.0018, ****p<0.0001.

(**C**) Representative TEM images of different fractions of the separated sEVs and extracellular particles secreted by senescent cells using iodixanol density gradient, demonstrating that fraction 10 contains the majority of the extracellular particles (n = 3 independent experiments).

(**D**) Representative TEM images and the corresponding quantification of the dsDNA-immunogold labeling of the isolated sEVP fractions secreted by senescent cells treated with MDM2 inhibitor or DMSO vehicle, demonstrating the presence of dsDNA on extracellular particles secreted by senescent cells. Data are presented as mean ± SD (n = 3 independent experiments). Statistical significance was determined using a two-sided unpaired t-test: **p =0.0068.

Supplemental Figure 1: Validation and characterization of CCF-specific secretion.

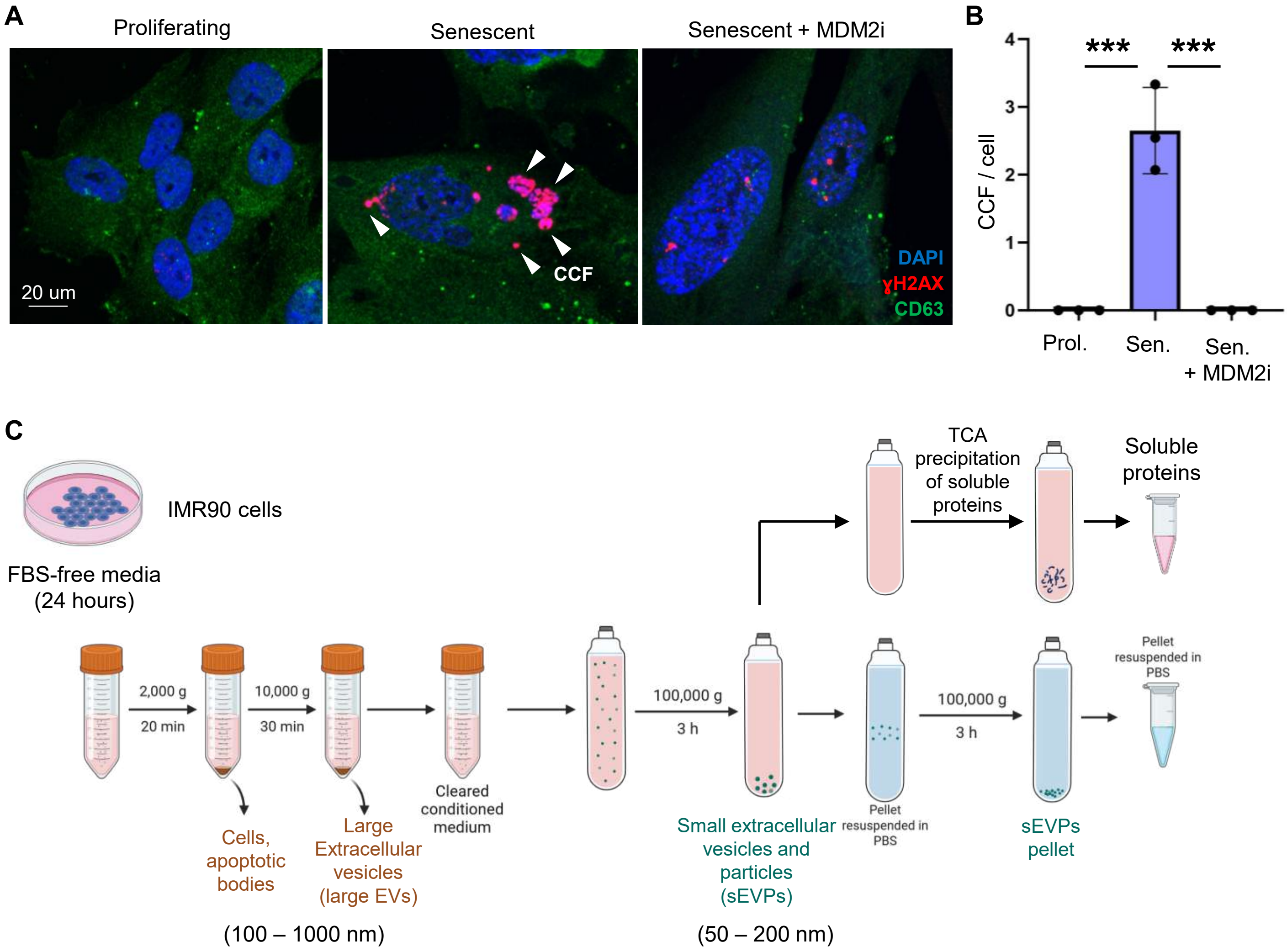

Supplemental Figure 2: Senescent cells upregulate the set of genes involved in endosomal pathways

A

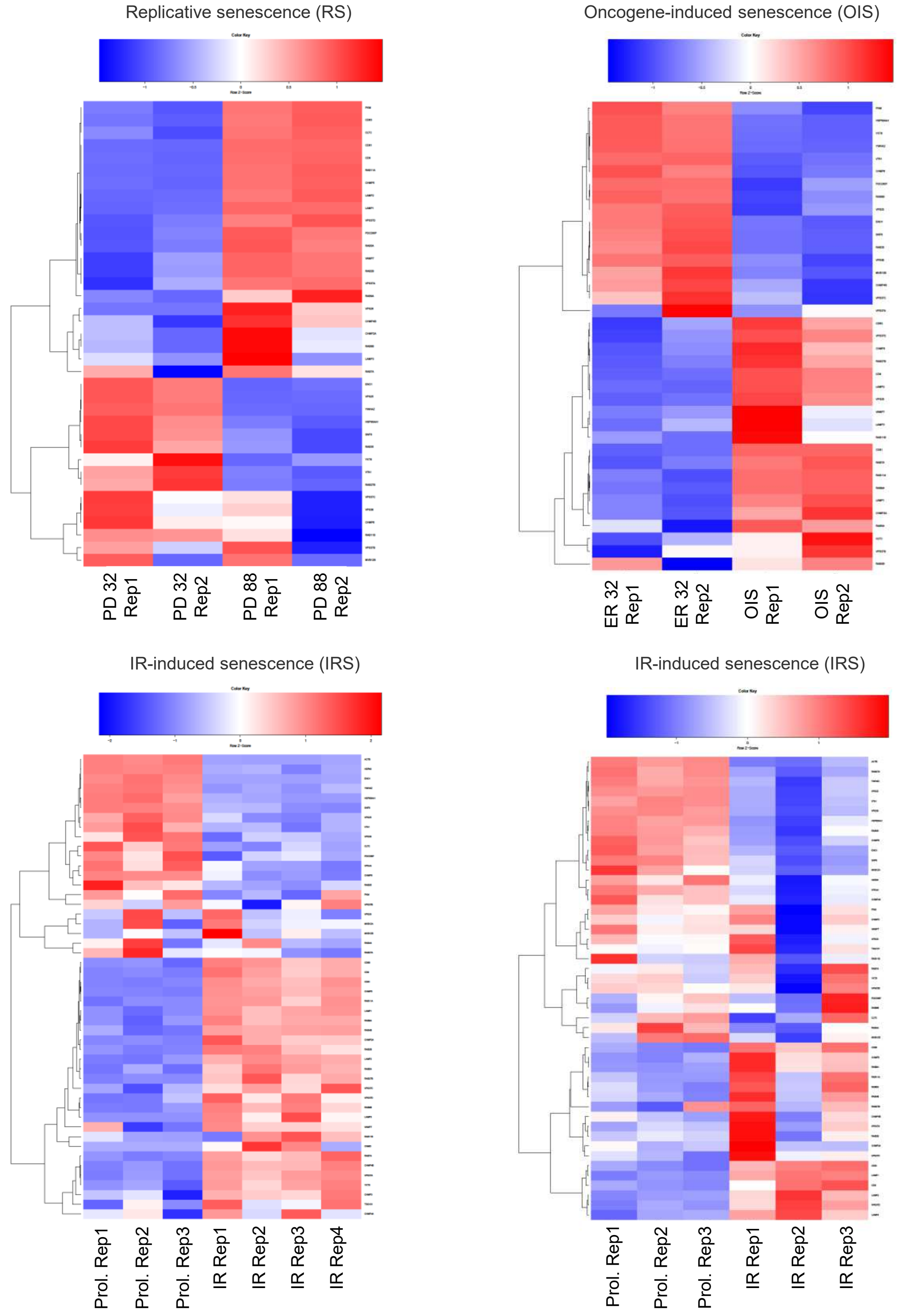

B

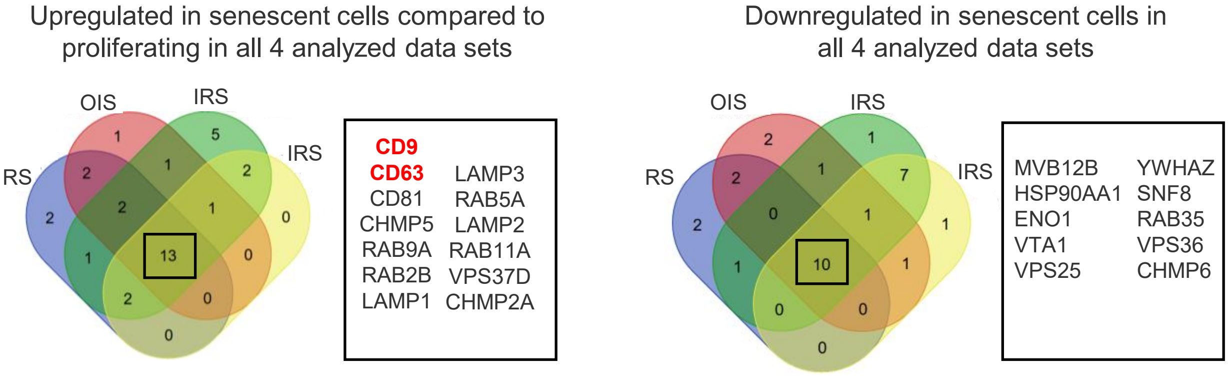

Supplemental Figure 3: Blocking intracellular degradation of CCFs by ATG16L1 knockout upregulates secretion of the components of CCFs

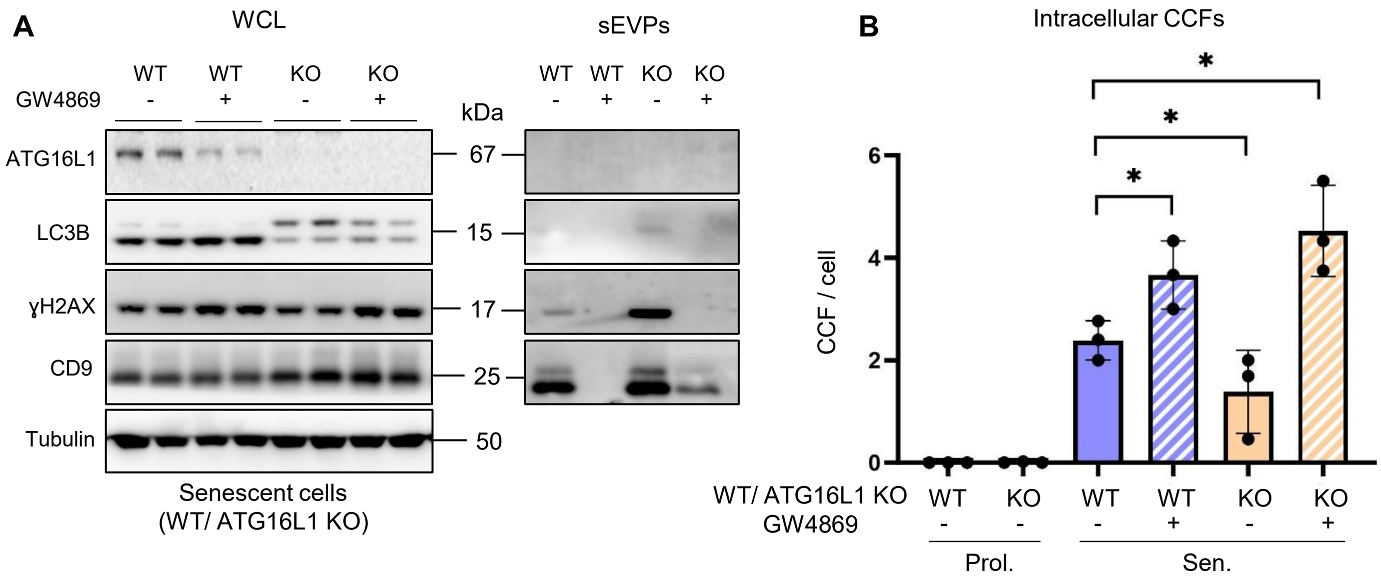

Supplemental Figure 4: Senescence alters the secretion profile of small extracellular vesicles and particles across cellular models, perturbation, and aging.

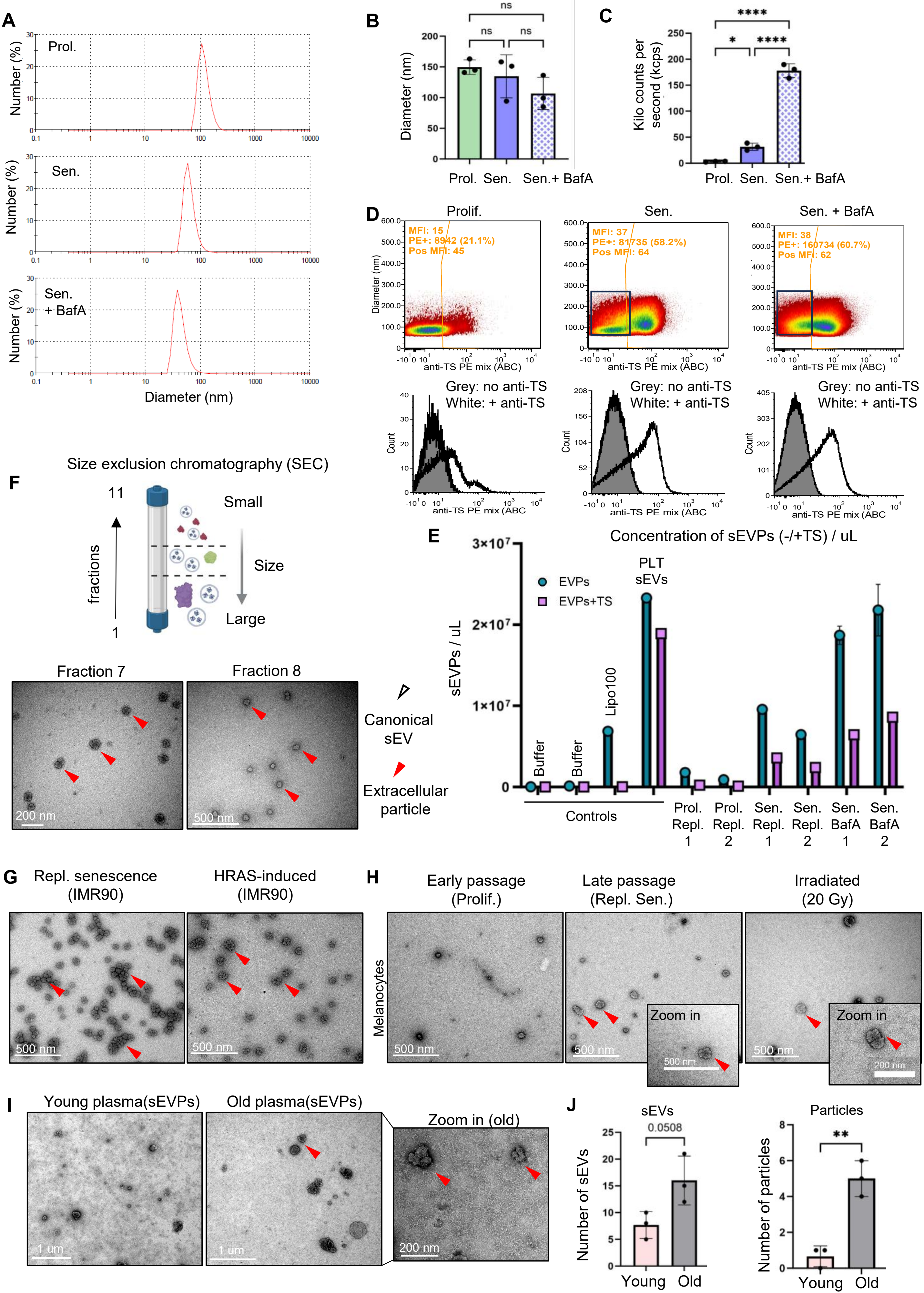

Supplemental Figure 5: Autophagy inhibition alters the secretion profile of senescent cells, enhancing the release of extracellular particles and associated dsDNA.

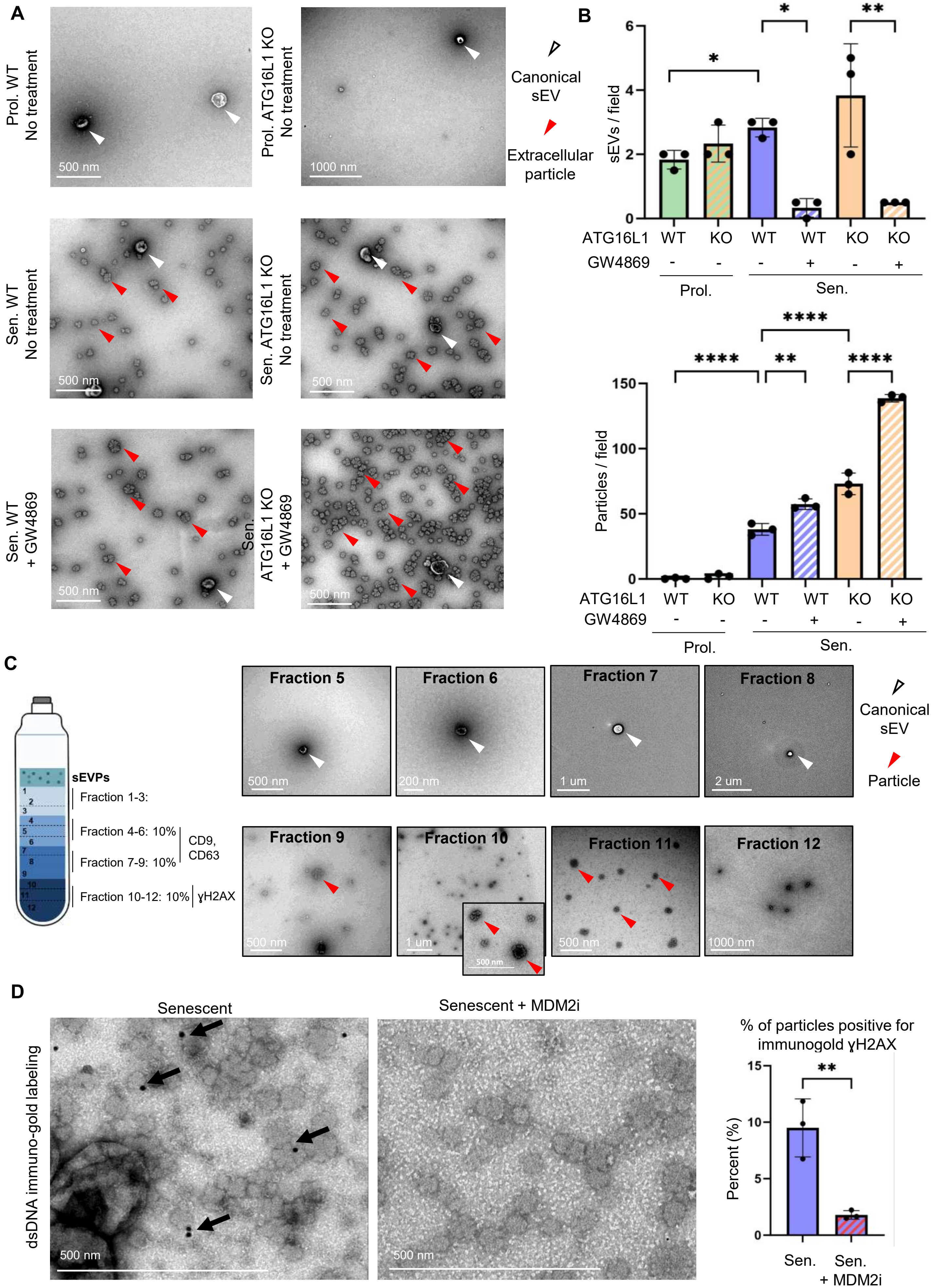

Supplement: Supplement 1 [file NIHPPrs8349343v1-supplement-1.pdf]
